# Widely Available Large Language Models Are Not a Reliable Source to Address Medical Treatment Recommendations of Patients After a First-Time Anteroinferior Shoulder Dislocation

**DOI:** 10.1016/j.asmr.2025.101216

**Published:** 2025-07-08

**Authors:** Elian Niklas Oudintsov, Soraya Bahlawane, Agahan Hayta, Cagman Seker, Doruk Akgun, David Alexander Back, Rony-Orijit Dey Hazra

**Affiliations:** Department of Shoulder and Elbow Surgery, Center for Musculoskeletal Surgery, Campus Virchow Klinikum, Charité – Universitätsmedizin Berlin, corporate member of Freie Universität Berlin and Humboldt-Universität zu Berlin, Berlin, Germany

## Abstract

**Purpose:**

To assess the ability of ChatGPT 3.5 to aid in the treatment planning process of first-time anteroinferior shoulder dislocation.

**Methods:**

Forty fictional patient cases were created varying in 15 different characteristics, whose distribution was randomized. Six orthopaedic surgeons (3 residents and 3 specialists in shoulder surgery) were then asked to determine the best treatment option for these patient cases. Their answers were compared with the treatment recommendations proposed by ChatGPT in 2 different sessions on the basis of preselected literature. To counteract the wide dispersion of responses, tendencies towards nonoperative, open surgical, or arthroscopic treatment were subsequently defined. The results were then analyzed descriptively.

**Results:**

The mean age of the fictional patients was 44 years (13-80 years), with 57.5% of the patients female. The agreement between the ChatGPT responses in the 2 sessions was 70.0%. In contrast, the 3 assistant physicians agreed with each other in 35% of all cases and the 3 specialists agreed in 32.5% of all cases. There was an exact match of 12.5% between the ChatGPT responses and all human assessments. In 65.0% of all cases, the physicians showed similar tendencies in their choice of therapy resulting in a 55.0% match between ChatGPT and the surgeons.

**Conclusions:**

There was no clear consensus regarding the treatment for first-time anteroinferior dislocations of the shoulder, neither among physicians nor with ChatGPT 3.5. However, ChatGPT 3.5 and physicians showed similar tendencies regarding the treatment in over half of the cases. Because of the inconsistent responses of ChatGPT 3.5, it cannot yet be considered as reliable tool for therapy planning.

**Clinical Relevance:**

ChatGPT 3.5, widely available and free of charge, is increasingly used in clinical settings. However, it’s crucial to highlight its limitations in treatment planning for pathologies, especially when there’s no clear consensus even among experienced surgeons.

There is no clear consensus on the optimal treatment strategy for a first-time anterior shoulder dislocation. A variety of treatments are discussed, ranging from nonoperative to surgical treatment. Specifically, arthroscopic labral repair and open or arthroscopic bony augmentations are addressed in the literature.^1^ Various patient-specific factors are weighted before making a decision. Mainly, next to imaging, gender and activity level lead to the choice of nonoperative or surgical therapy. Hence, men are approximately 3 times more likely to experience an acute shoulder dislocation than woman.^2^^,^^3^ Structural factors such as the degree of the glenoid defect, the extent of the Hill-Sachs and Bankart lesion, and associated additional soft-tissue injuries are described to be factors influencing decisions.^4^ In addition, the patient’s treatment goal must be considered, which can range from the ability to use the injured shoulder in daily activities to the expectation of a return-to-sport at a professional level.^5^

Given the large number of different factors that must be taken into consideration, the treatment decision should usually be made by an experienced orthopaedic surgeon. In the context of nowadays digital innovations, the question arises whether this decision-making process can be facilitated by using artificial intelligence (AI). Innovative solutions with the use of AI are currently emerging in almost every industry branch with essential impact in several fields such as informatics, technology, finance, and many more.^6^

Different large language models exist, with ChatGPT (OpenAI, San Francisco, CA), Google Gemini (formerly known as Google Bard) (Google LLC, Mountain View, CA), and the Microsoft Bing AI (Microsoft Corporation, Redmond, WA) being among the most known models. Like the other programs, ChatGPT is an advanced large language model (LLM) and has been accessible online since November 2022 with a well-known easy-to-use chat-function.^7^ “GPT” stands for “Generative Pre-trained Transformer“ technology, meaning that this AI has been pretrained with extensive datasets to carry out its function.^7^

Currently, the latest freely available version of ChatGPT 3.5 has not yet been used as a tool for treatment planning in orthopaedic surgery. An authors group of Garg et al.^8^ have illustrated the use of ChatGPT in the medical field in research. According to this study group, ChatGPT is not designed for treatment planning in general.

The purpose of this study was to assess the ability of ChatGPT 3.5 to aid in the treatment planning process of first-time anteroinferior shoulder dislocation. It was hypothesized that ChatGPT 3.5 as a LLM would be a reliable tool for treatment planning of first-time anteroinferior traumatic shoulder instability.

## Methods

### General Study Design

**Fig 1:**
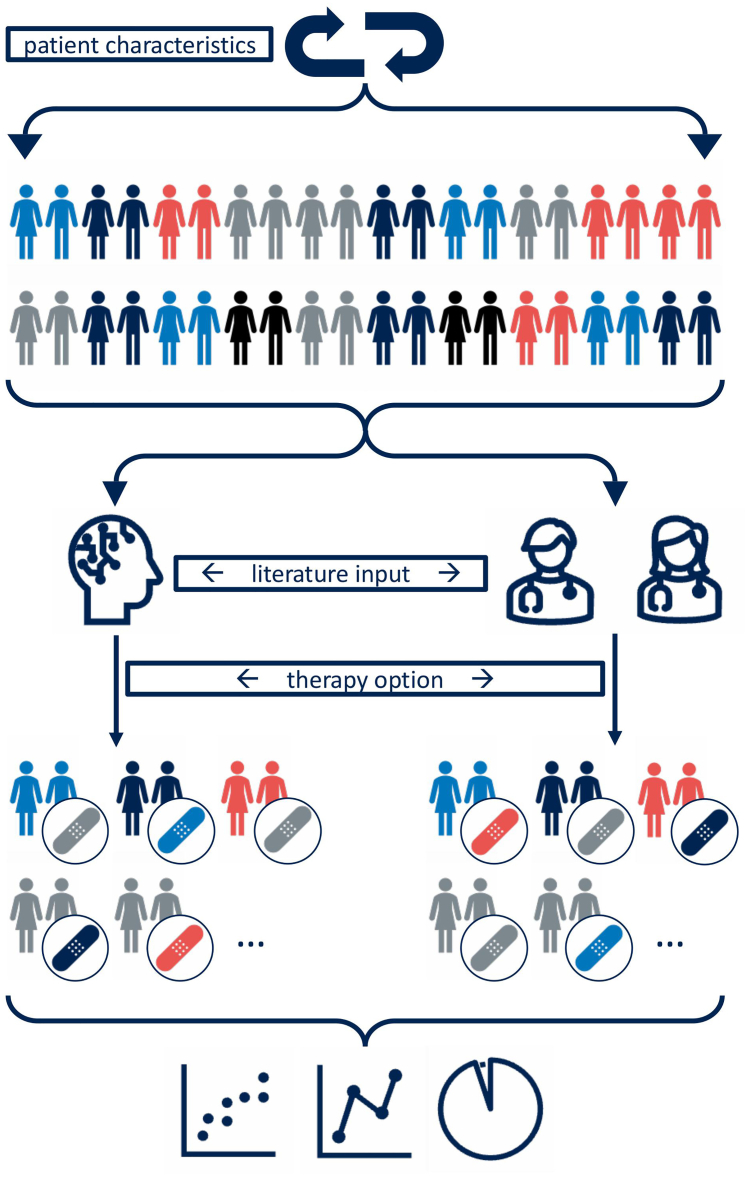
General study design.

Forty fictional patient cases were generated. Those cases were presented to ChatGPT and 6 orthopaedic surgeons. The surgeons consisted of 2 subgroups with either 3 residents or 3 specialists in shoulder surgery. The surgeons and the LLM were presented each case and were asked to select 1 of 5 given therapy options: nonoperative therapy; open surgical bony augmentation (Laterjet technique or J-Span technique); or arthroscopic approach (anterior stabilization with or without remplissage). The general study design is shown in Figure 1.

### Literature Search

To prevent the LLM from using fake or inaccurate sources, papers were chosen from scientific publications by a search of current literature available. The LLM was asked to use those papers only for answering the given questions (see to follow). Literature research was performed on PubMed using the strings “anterior shoulder dislocation” AND treatment” AND “algorithm” as well as “primary anterior shoulder dislocation” AND “treatment” to mimic the methods through which a physician seeking information for clinical decision making would gather information. The publications found were discussed and included by all 6 physicians. The inclusion criteria for these studies were the following: studies categorized with a Level of evidence of I or II; open-access texts; and studies were available in English language.

### Use of the LLM

The LLM product and version used in this study was ChatGPT 3.5, as it is widely available and free of charge. Because the LLM version used could only process up to 10,000 characters per request, only the introduction, results, and the discussion of the chosen papers were provided. To achieve a better comparability between the answers of the surgeons and ChatGPT, the LLM was used twice by 2 independent raters who asked the exact same questions. After obtaining all answers, tendencies toward nonoperative, surgical, or arthroscopic treatment were measured. A tendency was defined as given if there was a simple majority of responses in the subgroups. The answers and tendencies given by the physicians and the AI were compared in the according subgroups and among the other subgroups.

### Design of Patient Cases

Each of the 40 fictive cases contained 15 different characteristics such as age, gender, dominant arm, affected arm, level of physical activity, comorbidities, alcohol and drug use, course of accident, hypermobility, frozen shoulder, shoulder instability, bony defects, Hill-Sachs defects, and rotator cuff tears. The qualities of the characteristics were randomly defined by using the RANDBETWEEN-function in Microsoft Excel for Mac (Version 16.91, 2024; Microsoft, Redmond, WA) that generates a random integer number in a given range. After a first evaluation of all fictive patient cases, the characteristics were adjusted by the shoulder specialists to depict more realistic cases. Each fictional patient was assigned to an age-group: “12-18 years,” “19-40 years,” and “41-80 years.” The final qualities and probabilities are shown in Table 1. To make the patient cases accessible for the LLM and the physicians, the given characteristics per fictional case were summarized in case reports in a standardized way.

**Table 1:** Incidence-Rates and Expected Incidence Rates of All Patient Characteristics

Quality	Incidence Rate, %	Probability/ Expected Rate, %	Comment
Gender			
Female	57.5	50.0	
Male	42.5	50.0	
Age			
12-80 yr			
Dominant arm			
Left arm	42.5	50.0	
Right arm	57.5	50.0	
Course of accident			
Traumatic	42.5	50.0	
Atraumatic	57.5	50.0	
Affected arm			
Left arm	50.0	50.0	
Right arm	50.0	50.0	
Level of physical activity			
No physical activity	22.5	25.0	
Hobby sport	27.5	25.0	
Competitive sport	20.0	25.0	
Overhead sport	30.0	25.0	
Comorbidity			
No comorbidity	57.5	55.6	
Diabetes mellitus type I or II	10.0	11.1	
Diabetes mellitus type I or II, high blood pressure	12.5	11.1	
Diabetes mellitus type I or II, high blood pressure, bleeding tendency	7.5	11.1	
Diabetes mellitus type I or II, high blood pressure, bleeding tendency, stroke, or myocardial infarction	12.5	11.1	
Alcohol consumption			
None	20.0	25.0	Only patients with an age of 16 years or older could have alcohol consumption.
Low alcohol consumption	17.5	25.0	
Moderate alcohol consumption	30.0	25.0	
High alcohol consumption	27.5	25.0	
Drug use			
No	52.5	50.0	Only patients with an age of 16 years or older could have drug use.
Yes	47.5	50.0	
Hypermobility			
No (Beighton-score <4)	50.0	50.0	
Yes (Beighton-score ≥4)	50.0	50.0	
Frozen shoulder			
No	60.0	50.0	
Yes	40.0	50.0	
Feeling of instability			
No	50.0	50.0	
Yes	50.0	50.0	
Imaging: glenoid defect			
No glenoid defect	55.0	50.0	
Small glenoid defect	45.0	50.0	
Imaging: Hill-Sachs defect			
No Hill-Sachs defect	55.0	33.3	Patients without a glenoid defect have no Hill-Sachs defect with a probability of 100%.
Small Hill-Sachs defect	30.0	33.3	Patients with a glenoid defect could have a small Hill-Sachs defect with a probability of 50.0%
Big Hill-Sachs defect	15.0	33.3	
Imaging: rotator-cuff injury			
No	50.0	50.0	
Yes	50.0	50.0	

### Statistical Analysis

The fictional patient cases and the obtained answers were analyzed descriptively using Microsoft Excel for Mac (version 16.91; 2024 Microsoft) and IBM SPSS Statistics (version 28.0.0.0; IBM Corp., Armonk, NY). The collected data were checked for normal distribution using the Shapiro-Wilk test. The significance level was set at *P* = .05. The Man-Whitney *U* test was used for comparing 2 independent, nonparametric distributed variables between groups. The Kruskal-Wallis test was used if there were more than 2 independent, nonparametric distributed variables.

## Results

### Literature Research

After the literature study performed on December 10, 2023, 38 publications were included. Following the given criteria, 33 publications were excluded, leaving 5 publications for being presented to the LLM.^9^, ^10^, ^11^, ^12^, ^13^

### Patient Cases and Characteristics

The mean age of the 40 fictional cases was 43.5 years, ranging from 13 to 80 years. The age was not distributed normally, *P* = .004. The assigned age-groups were therefore also not distributed normally, *P* < .001. Only 4 patients belonged to the age group ranging from 12 to 18 years, whereas 16 patients were assigned to the age group ranging from 19 to 40 years and 20 patients are located between 41 and 80 years. In this group of fictional cases 57.5% were female and 52.5% of all cases were left-handed. The left and right side were affected equally. In total, 77.5% of the fictional patients did any kind of sport; 42.5% had 1 or more comorbidities; 80.0% of the patients drank alcohol; and 47.5% had a positive drug history. In total, 42.5% of the shoulder dislocation were of traumatic origin; 50% of the patients were hypermobile, and 40.0% of the patients experienced frozen shoulder syndrome. One half of the patients mentioned shoulder-instability. Radiologic imaging showed that 45.0% of the fictional cases presented with a bony Bankart defect. An equal number of patients also had a Hill-Sachs defect. In 50.0% of the cases the rotator cuff was affected. None of the aforementioned characteristics was distributed normally. The exact breakdown of characteristics can be seen in Table 1.

### Consistency in Given Answers

The treatment plans selected by the 3 residents aligned in 35.0% of all cases. The shoulder specialists were unanimous in 32.5%. All physicians showed an exact consensus in only 15.0% of all 40 fictional cases. On the other side, the answers given by the LLM after insertion by the 2 raters were consistent in 28 of 40 fictional cases (equal to 70.0%). ChatGPT and the residents as well as ChatGPT and the shoulder specialists agreed in 20.0% of the cases. The total alignment between all physicians and the LLM therefore amounted to 12.5%.

### Consistency in Determined Treatment Tendencies

In 65.0% of the cases (n = 26) a treatment tendency for all physicians could be determined. The LLMs’ tendencies corresponded in 55.0% of the cases with the determined physicians' tendencies. For further detail the matches between all different subgroups are showed in the following table (Table 2).

**Table 2:** Percentage Of Matching Treatment Tendencies Between Different Subgroups

	Residents (n = 3)	Shoulder Surgeon (n = 3)	ChatGPT (n = 2)
Residents (n = 3)			
Shoulder surgeon (n = 3)	65.0%		
ChatGPT (n = 2)	55.0%	55.0%	70.0%

### Treatment Tendencies in Relation to Specific Patient Characteristics

After calculating the treatment tendency with respect to all physicians, older patients were assigned significantly more often to nonoperative treatment (70.0% of all cases older than the age of 40 years), whereas the younger patients received more often a surgical treatment plan, *P* < .001. Female patients were more often assigned to a nonoperative treatment (n = 13), whereas male patients were recommended an arthroscopic approach (n = 10) more often, *P* = .024. Patients with a rotator-cuff injury were significantly more often assigned to an arthroscopic treatment (n = 15) by all physicians, *P* = .005. ChatGPT, in contrast, assigned significantly more patients with an alcohol consumption to an operative treatment (n = 17), *P* = .042. A detailed breakdown of statistical significances of all treatment tendencies in relation to evaluated patient characteristics is shown in Table 3.

**Table 3:** Statistical Significances of All Treatment Tendencies in Relation to Patient Characteristics

Characteristic	Residents (n = 3)	Shoulder Surgeons (n = 3)	All Physicians (n = 6)	ChatGPT (n = 2)
Age	**.043**	**<.001**	**<.001**	.978
Gender	**.013**	**.978**	**.024**	.516
Level of physical activity	.750	.554	.679	.406
Comorbidity	.173	.487	.685	.914
Alcohol use	1.000	.403	.607	**.030**
Drug use	.789	.851	.748	.270
Course of accident	.787	.978	.356	.871
Hypermobility	**.030**	1.000	.369	.192
Frozen shoulder	**.027**	.838	.569	.774
Shoulder instability	.602	.602	.445	.174
Bony Bankart defects	.600	.717	.757	.180
Hill-Sachs defects	.778	.347	.686	.323
Rotator-cuff tears	**.030**	**.030**	**.005**	.355

NOTE. *P* < .05 was defined as significant. Values in bold are statistically significant.

## Discussion

On the basis of the results of this study, the most important finding is that ChatGPT 3.5 did not prove to be a reliable source to address medical treatment recommendation of patients after a first-time anteroinferior shoulder dislocation in the given setting. AI and especially large language models are increasingly gaining importance in different areas in medicine.^14^ However, this tendency does not affect all areas in the same ways and is rather new in shoulder surgery. Although medical databases listed only 1 manuscript in 2022 and 5 in 2023, there has been increased publication rate of 420% (21 articles) in 2024. Most publications focus on the use of AI on imaging.^15^^,^^16^ The question arises if LLM can help the established shared decision making between a shoulder specialist and the patient.

The presented study revealed an agreement between the ChatGPT responses in the 2 independent sessions of 70.0%. This is in line with recently published literature. A study performed by Knee et al.^17^ examined the consistency of ChatGPTs` answers regarding distal fractures of the wrist and described an approximate agreement of 70.0% in 2 independent sessions. Likewise, Nwachukwu et al.^18^ published concordant response in 70.3% in 4 different LLMs regarding rotator cuff tears and anterior crucial ligament tears. This inconsistency by LLMs may be explained by their structure itself. ChatGPT is based on machine-learning, analyzing, and scanning through its database to find an answer suitable for a specific question and context, leading to different answers, depending on individual interpretation of the given data and on the basis of probabilities chosen by the LLM within a specific spectrum.^7^ Slightly different phrasing could alter the output and consequently lead to different recommendations.^19^ Furthermore, authors also described a so-called “memory fatigue” for ChatGPT with questions being asked earlier on a chat were found to have a better response quality.^20^ A possible reasoning could be the use of so-called tokens as small units of text, which can be processed by the LLM. Because a context window is limited to a specific number of tokens, the quality of answers might decrease in an ongoing chat history.^7^^,^^21^

In addition, the here gained data showed similar tendencies of the physicians in their therapy choices in 65.0% of all cases. This may be seen as being in contrast to current literature, where, e.g., an article by the Neer circle of the American Shoulder and Elbow Surgeons achieved a consensus in 5.0% of 162 cases.^22^ Nonetheless, a high rate of similarity in the given answers between different physicians may be explained by the study design with all surgeons working at the same unit with uniformed therapy regimes. However, more experienced physicians seemed to prefer a nonoperative treatment plan for patients older than the age of 40 years. It could be shown that the physicians take the several patient characteristics into account. Especially, age and rotator-cuff-injuries seem to be predominant factors for decision making. Physicians tended to treat patients younger than 30 years old and patients with rotator-cuff-tears operatively whereas ChatGPT seemed to prefer patients with alcohol consumption for an operative treatment.^23^

A further outcome of this study was, that tendencies offered by ChatGPT and the surgeons matched in 55% of the cases. This can be seen as in line with Agharia et al., who reported an alignment of ChatGPT 3.5. with orthopaedic surgeons treatment suggestions for different injuries (such as foot, ankle, hand, knee and sports injuries, pediatric, reconstruction, shoulder and elbow and spine injuries, and trauma) in 40.2%. Interestingly, ChatGPT 4 already achieved a higher congruence with 68.0%.^24^

In this context, a recent analysis of the answers of 4 LLMs to the 23 most frequently asked questions of patients concerning anteroinferior shoulder instability should be mentioned. The study group could present a high LLM quality in answers but also the necessity of a high reading level to understand the answers given.^25^ This might limit the applicability of LLMs to patients with a broad background on knowledge.

Regarding the accuracy of ChatGPT in the here presented study, the findings go along with a study that analyzed the suggestions of ChatGPT, Google Bard and Microsoft Bing for treatment and diagnostics of 50 fictive hematology patient cases.^26^ ChatGPT showed best results, before Bard and Microsoft Bing with an accuracy of 3.15 of 5.

### Limitations

This study has several limitations that should be considered when interpreting the data. The main drawback may be seen in the chosen LLM itself. ChatGPT obtains its information from a database that received its last update in January 2022. Therefore, ChatGPT cannot use any information that were published after the last update. For ChatGPT to still operate properly and not give inaccurate information it was necessary to provide specific resources. That limits its pool of information. Furthermore, ChatGPT can only process up to 10,000 characters per message, which makes it not possible to provide the full article. Therefore, only the introduction and the discussion of the articles were provided. Another limitation is the design of patient cases. The patient population was designed randomly, which does not reflect the general population. Therefore, the group of young patients is clearly underrepresented. Epidemiologic values were not considered because of the lack of such information. More standardized cases might have to be used in further studies. Finally, the number of participants can also be listed as a limiting factor. With a larger sum of participants, a greater statistical significance could be achieved and the distribution of the given answers could be minimized.

## Conclusions

There was no clear consensus regarding the treatment for first-time anteroinferior dislocations of the shoulder, neither among physicians nor with ChatGPT 3.5. However, ChatGPT 3.5 and physicians showed similar tendencies regarding the treatment in more than half of the cases. As a result of the inconsistent responses of ChatGPT 3.5, it cannot yet be considered as reliable tool for therapy planning.

## Disclosures

The authors declare the following financial interests/personal relationships which may be considered as potential competing interests: D.A. reports consulting or advisory, funding grants, and speaking and lecture fees from Charité University Hospital Berlin. All other authors (E.N.O., S.B., A.H., C.S., D.A.B., R-O.D.H.) declare that they have no known competing financial interests or personal relationships that could have appeared to influence the work reported in this paper
